# C-reactive protein and risk of ovarian cancer: A systematic review and meta-analysis

**DOI:** 10.1097/MD.0000000000007822

**Published:** 2017-08-25

**Authors:** Jing Li, Xuedan Jiao, Zhongfu Yuan, Haifeng Qiu, Ruixia Guo

**Affiliations:** a Department of Oncology, The First Affiliated Hospital of Zhengzhou University, Zhengzhou, Henan Province; b Reproductive Center, Department of Obstetrics and Gynecology, Sun Yet-Sen Memorial Hospital, Guangzhou, Guangdong Province; c Department of Obstetrics and Gynecology, The First Affiliated Hospital of Zhengzhou University, Zhengzhou, Henan Province, China.

**Keywords:** C-reactive protein, meta-analysis, ovarian cancer

## Abstract

**Background:**

Although several studies have suggested an association between elevated C-reactive protein (CRP) and ovarian cancer risk, others have yielded contradictory results. To address this issue, we conducted a meta-analysis.

**Methods:**

Studies were identified by searching PubMed and EMBASE up to July 2017 without language restrictions. Six case-control studies and 1 cohort study were included, including 1898 ovarian cancer cases. Pooled risk estimates were generated by using the fixed-effect model or the random-effect model based on the heterogeneity between studies.

**Results:**

As our data shown, the combined ORs were 1.04 (95%CI: 0.90–1.21) and 1.34 (95% CI: 1.06–1.70) for the risk in the second and third tertiles of CRP with those in the bottom tertile, respectively. Subgroup analysis showed that with respect to the top tertile of CRP level, the association was significant for studies obtaining CRP from serum (OR=1.99; 95% CI: 1.30–3.07), conducted in the USA (OR = 1.41; 95% CI: 1.15–1.72), using high-sensitivity immunotubidimetric assay (OR = 1.37; 95% CI: 1.14–1.64), using Hs-CRP (OR = 1.46; 95% CI: 1.21–1.75) and with follow-up period longer than 10 years (OR = 1.41; 95% CI: 1.18–1.70).

**Conclusion:**

Collectively, our findings propose that serum CRP levels may serve as an indicator of ovarian cancer risk. Further studies are needed to definitively identify the role of CRP in the etiology of ovarian cancer.

## 1 Introduction

Worldwide, ovarian cancer is the most lethal gynecologic cancer.^[1]^ There is an estimated 21290 new cases and 14180 new deaths caused by this malignancy in the United States in 2015.^[2]^ Due to the limited knowledge about the etiology of ovarian cancer, there are currently no effective approaches for detection and treatment. Thus, more than 70% of ovarian cancer patients are already at advanced stages when diagnosed with significant morbidity and mortality.^[3,4]^ During the past half century, a large amount of evidence has linked inflammation with the onset of ovarian cancer; studies have found that pelvic inflammation and endometriosis are notably associated with an increased risk of ovarian cancer.^[5–7]^ In turn, researchers suggest the use of anti-inflammation drugs may reduce the incidence of ovarian cancer.^[8]^

The C-reactive protein (CRP) is a highly sensitive and widely used systematic marker of inflammation, produced primarily by hepatocytes along with other acute-phase proteins and released into circulation in response to tissue injury and inflammation.^[9]^ Accumulating epidemiologic studies have demonstrated the association between elevated CRP levels and the risk of epithelial cancers, such as liver, lung, colorectal, endometrial, and breast cancers.^[10–13]^ However, data on the association between CRP and ovarian cancer risk are sparse and inconsistent.

Several prospective studies have suggested the positive association between the pre-diagnostic CRP level and the ovarian cancer risk, with the odds ratio ranging from 1.09 to 2.33 for the top tertile compared with the bottom tertile.^[14–19]^ However, different results were obtained by Lundin et al,^[18]^ who observed no overall correlation between the 2 parameters, but a strong positive association for women with CRP concentrations greater than10 mg/L, using American, Swedish, and Italian subjects. Similar results were also found by Ose and colleagues,^[15]^ which is the most recent and largest prospective study by far, with a total of 754 ovarian cancer cases and 1479 normal controls. As there are conflicting results on the association between the CRP level and the ovarian cancer risk, we conducted the meta-analysis to systematically assess the relationship between increased CRP levels and the ovarian cancer risk.

## 2 Materials and methods

### 2.1 Publications selection

A comprehensive literature search and review up to July 2017 was performed through PubMed and Excerpta Medica database (EMBASE) to identify relevant articles. Search terms included “C-reactive protein” or “C-reactive protein” or “CRP” along with terms “ovarian cancer” or “ovarian carcinoma.” Additional relevant references cited in retrieved articles were also evaluated.

### 2.2 Inclusion and exclusion criteria

All papers were reviewed by 2 authors independently. Uncertainties and discrepancies were resolved by consensus after discussing with a senior researcher. No language restrictions were imposed. All studies included in the final meta-analysis satisfied the following criteria: (1) case-control or cohort study design; (2) reporting results on blood CRP levels; (3) ovarian cancer incidence as the outcome of interest; (4) reporting odds ratio (OR) in case-control studies or relative risk (RR) with their corresponding 95% CI (or sufficient data to calculate these effect measures). If the study was reported in duplication, the one published later or provided more detailed information was included. Review and editorials were included if they met the above 4 criteria. Abstracts and full texts without original data even after having contacted the authors were excluded.

### 2.3 Data extraction

Two of the authors performed the data extraction from each article and resolved the discrepancies in consensus. For studies meeting inclusion criteria, a standardized data extraction form was used to extract the following data: the first author's name, year of publication, study design, country of origin, cohort or case-control study name, period of enrollment, the number of participants, cancer cases and controls, CRP measurement methods, CRP markers, the clinical outcome assessment methods, OR or RR estimates with corresponding 95% CIs for CRP as a continuous variable or at least 3 categories of CRP levels. For each study, we extracted the risk estimates that were adjusted for the greatest number of potential confounders.

The OR or RR in the natural log scale change in increased tertile of CRP level with their standard error (SE) was used to compute the combined OR of elevated CRP level and the risk of ovarian cancer. For studies that reported 95%CIs of OR instead of SE of log(OR), we first estimated the lower and upper bounds of SE by using the inversed function for 95% CI. The SE was then approximately estimated as the squared root of the product of the lower and upper bounds obtained above. Weighted estimation with inverse-variance weights was used to assess the overall averaged risk of increased CRP level. Fixed-effect and random-effect models were selected for meta-analysis based on the extent of heterogeneity between publications. The Cochrane *Q* test (*P* < .05 indicated a high level of statistical heterogeneity) and *I*^2^ (values of 25%, 50% and 75% corresponding to low, moderate, and high degrees of heterogeneity, respectively) was used to evaluate the heterogeneity between eligible studies, which test total variation across studies that was attributed to heterogeneity rather than to chance. As substantial heterogeneity was detected between studies, the random-effect model that allows for possible variations of risks was adopted. The restricted maximum-likelihood estimator (REML) was used to account for the amount of heterogeneity and fitted the random-effect model.

Subgroup analysis for increased CRP level and their risk of ovarian cancer were subsequently carried out by the country of origin, CRP markers, CRP assay methodology, sample source, follow-up period, and the adjusted variables of age, BMI, smoking, parity, nonsteroidal anti-inflammation drugs (NSAIDs) use, hormone therapy (HT) use, and oral contraceptive (OC) use, respectively. Sensitivity analysis was also conducted to assess the influence of each individual study on the strength and stability of the meta-analysis results. To show each study's independent impact on the combined effect, 1 study in the meta-analysis was excluded at each time and the combined effect was recalculated. Funnel plots and 2 widely used statistical tests including Begg's adjusted rank correlation test and Egger's regression test on asymmetry of funnel plot were performed to test any existing publication bias.

### 2.4 Statistical analysis

All analyses were performed using the Meta-Analysis Package for R (“metaphor” package version 1.9–7) under R statistical environment (version 3.1.0).^[20]^ A 2-tailed *P* < .05 was considered as statistically significant.

## 3 Results

### 3.1 Literature search

**Figure 1:**
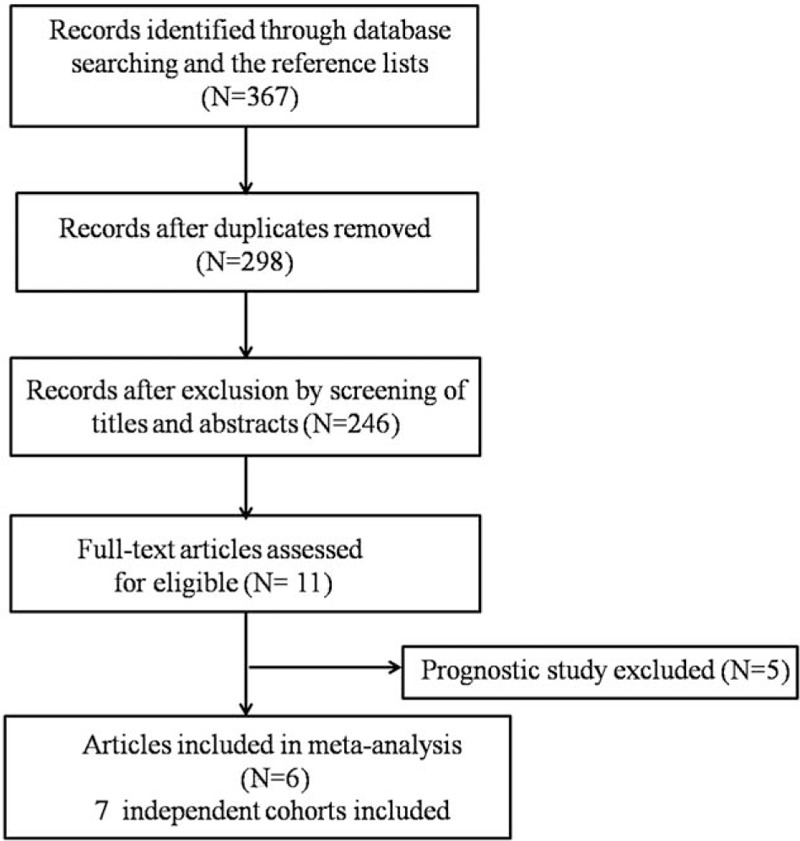
Flow diagram of systematic literature search.

A total of 367 publications were retrieved from the initial literature search, of which 52 studies were considered potentially valuable after reviewing the titles and abstracts. The full text was then retrieved for detailed evaluation. After further investigating the studies, 5 were screened out due to prognostic studies. Finally, 6 studies were eligible for meta-analysis, including 6 independent case-control studies and 1 cohort study. The flow chart of the search process is shown in Fig. 1.

### 3.2 Characteristics of the selected studies

The characteristics of the included 6 case-control studies and 1 cohort study are summarized in Table 1. They were published from 2007 to 2017 and including a total of 1898 ovarian cancer cases. Among these studies, 3 were conducted in the United States, 3 in Europe, and 1 in both. The study design of 6 studies was a nested case-control study and 1 was a cohort study. Cancer diagnoses were confirmed in 2 studies by linkage to both cancer registries and pathology reports, 2 by cancer registry, 2 by medical records, and 2 was not provided. CRP was measured in 4 studies by high-sensitivity CRP marker, 4 by high sensitivity immunotubidimetric assay, 1 by enzyme-linked immunosorbent assay (ELISA), 1 by high sensitivity immunoassay, and 1 by Luminex bead-based assay. All the 7 cohorts were adjusted for age, smoking, NSAIDs use, hormone replacement therapy (HRT) use, and oral contraceptive (OC) use; 6 were adjusted for BMI and parity status.

**Table 1:**
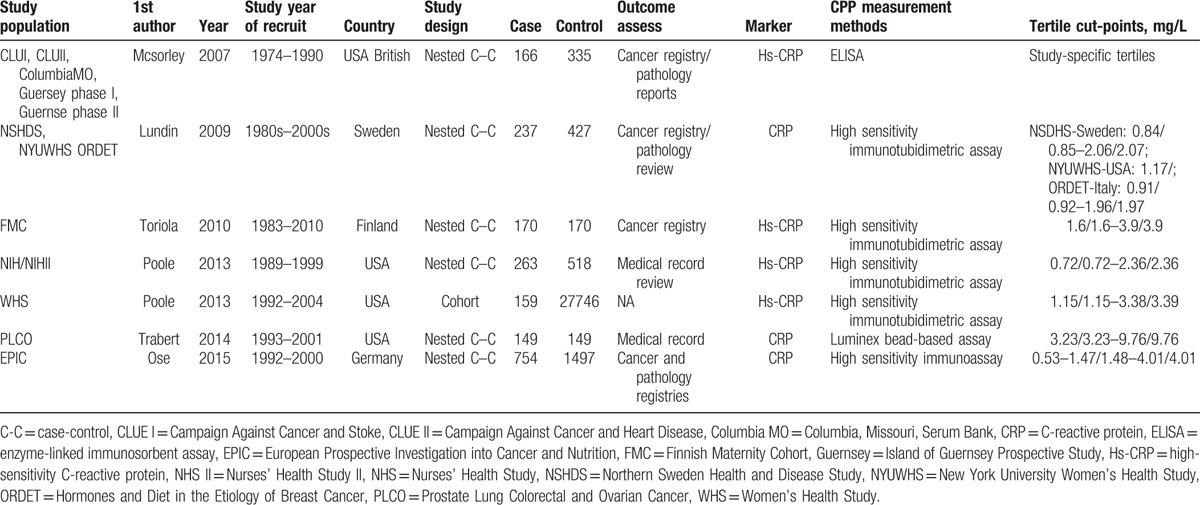
Characteristics of the included studies.

### 3.3 Association between the CRP level and elevated ovarian cancer risk

**Figure 2:**
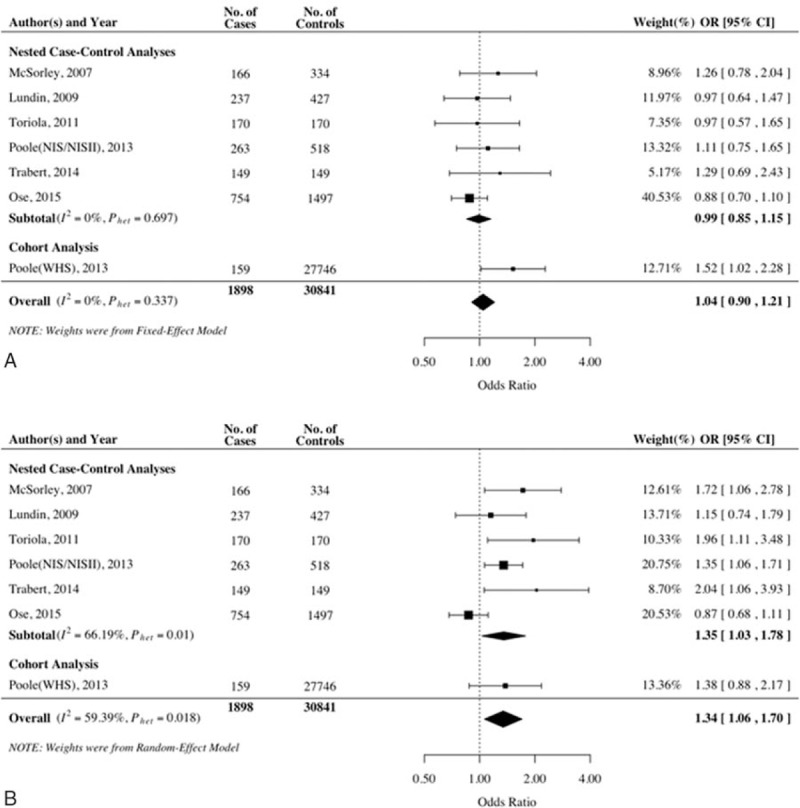
Forest plot for the association between the CRP level and the ovarian cancer risk. (A) The second tertile of CRP level; (B) the third tertile of CRP level. CRP = C-reactive protein.

**Figure 3:**
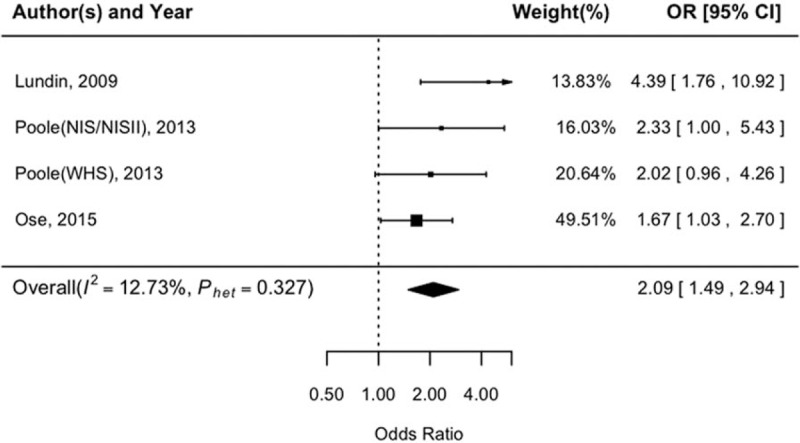
Odds ratio of ovarian cancer among women with a CRP level greater than 10 mg/L. CRP = C-reactive protein.

The reported covariates-adjusted OR for each study and the overall risk estimation for CRP are shown in Fig. 2. When comparing women in the second tertile of CRP with those in the bottom tertile, the combined ORs were 1.04 (95%CI: 0.90–1.21). No significant heterogeneity was found in this scenario (*P*_heterogeneity_ = .337, *I*^2^ = 25.97%). As comparing women in the top tertile of CRP with those in the bottom tertile, the pooled estimates of ORs was 1.34 (95% CI: 1.06–1.70). Heterogeneity across the studies was significant (*P*_heterogeneity_ = .018, *I*^2^ = 59.39%). Consistent results were found by using only 6 case-control studies (OR = 0.99, 95%CI: 0.85–1.15, and OR = 1.35, 95%CI: 1.03–1.78, for the second and top tertiles, respectively). In addition, 4 studies reported the significant association between higher CRP levels (>10 mg/L) and ovarian cancer risk in consensus. As shown in Fig. 3, a CRP level greater than 10 mg/L presented a remarkably increased risk for ovarian cancer (ORs = 2.09; 95% CI: 1.49–2.94; *P*_heterogeneity_ = .327, *I*^2^ = 12.73%).

### 3.4 Subgroup analysis

To explore the heterogeneity among studies evaluating CRP levels and ovarian cancer risk, we performed subgroup analysis (Table 2). The association of CRP level with ovarian cancer risk did not differ by any of the stratified subgroups. With respect to the third tertile of CRP level, the association was stronger in the group of sample sources from serum (OR = 1.99; 95% CI: 1.30–3.07) than those from plasma (OR = 1.36; 95% CI: 1.10–1.68), although this was not statistically significant. The combined OR for ovarian cancer was significant for studies conducted in the USA (OR = 1.41; 95% CI: 1.15–1.72), but not significant in Europe (OR = 1.18; 95% CI: 0.76–1.85). Stratifying results by CRP assay methodology, CRP markers and follow-up period showed that high-sensitivity immunotubidimetric assay, Hs-CRP, and follow-up period longer than 10 years had significant associations, whereas the association disappeared for CRP levels measured by other assays, common CRP, and a follow-up period shorter than 10 years. In addition, the associations were significant for studies adjusted for smoking, NSAIDs, and OC use, whereas no significance was found for those adjusted for age and HT use.

**Table 2:**
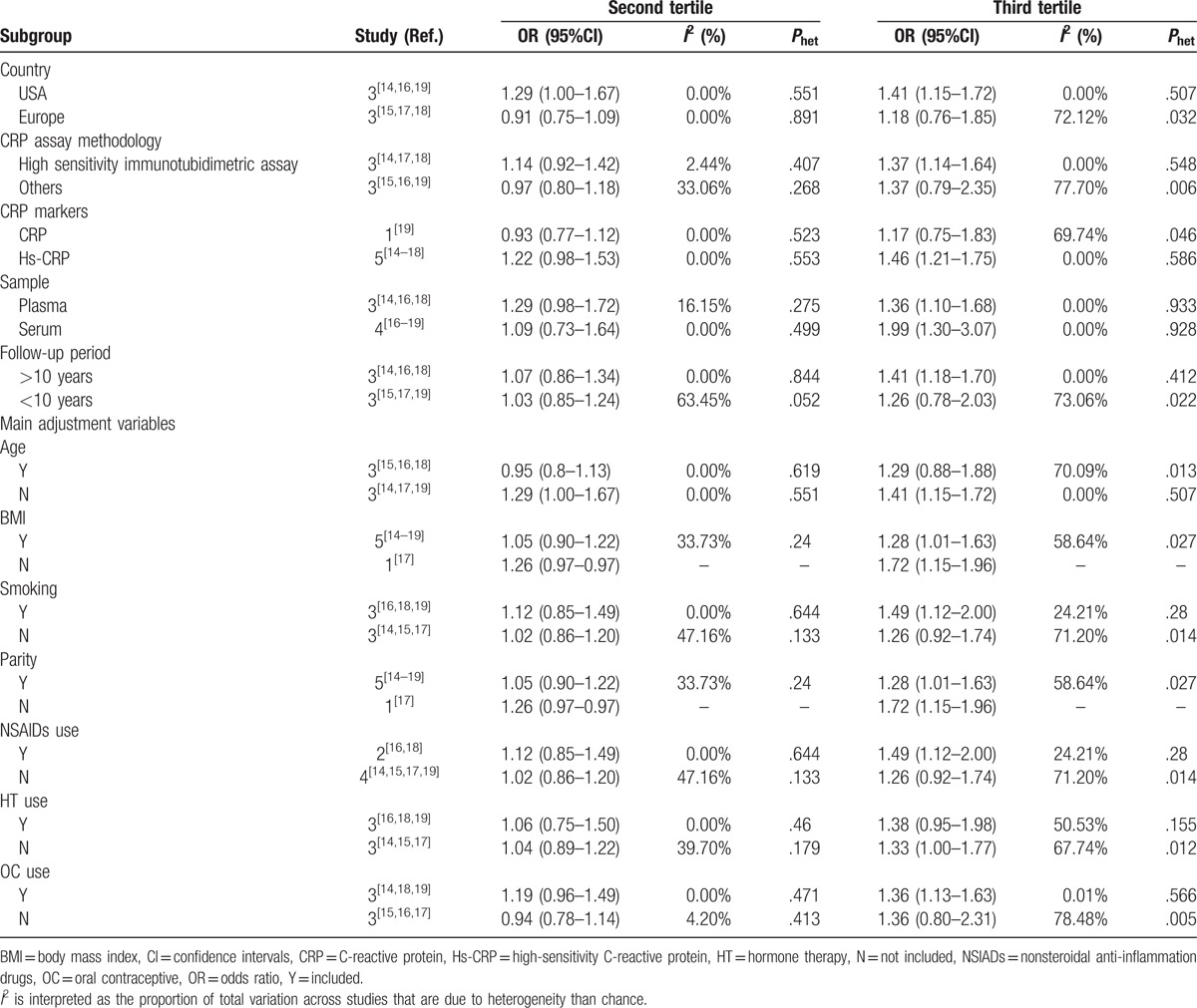
The results of subgroup-analyses.

### 3.5 Influence analysis of individual studies

**Figure 4:**
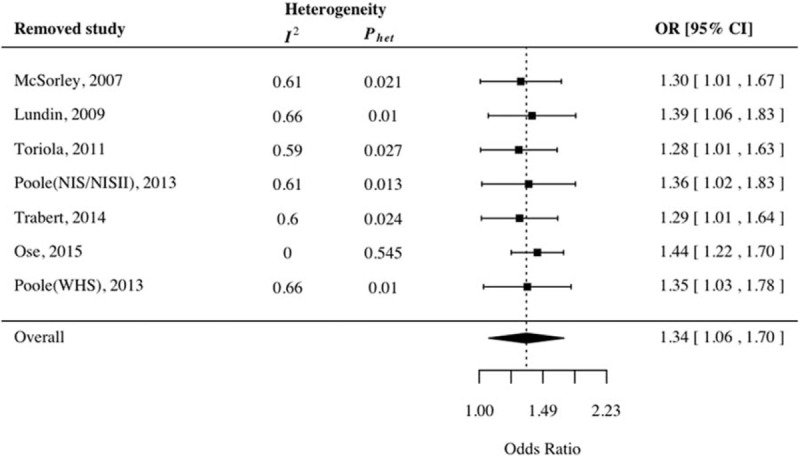
Influence analysis for omitting individual study on the summary odds ratio.

Sensitivity analysis was also performed to address the potential heterogeneity due to the quality of the included studies and their impact on the combined effect (Fig. 4). The combined OR in CRP levels ranged from 1.28 (95% CI: 1.01–1.63) to 1.44 (95% CI: 1.22–1.70). No substantial departure was found on these combined effects. A consistent amount of heterogeneities (ranged from 0.59 to 0.66) were detected for omission of any of the individual studies except that for Ose and colleagues’ study (OR = 1.44, 95% CI: 1.22–1.70; *P*_heterogeneity_ = .545, *I*^2^ = 0%), which indicated that this particular study contributes to a great amount of the heterogeneity seen.

### 3.6 Publication bias

**Figure 5:**
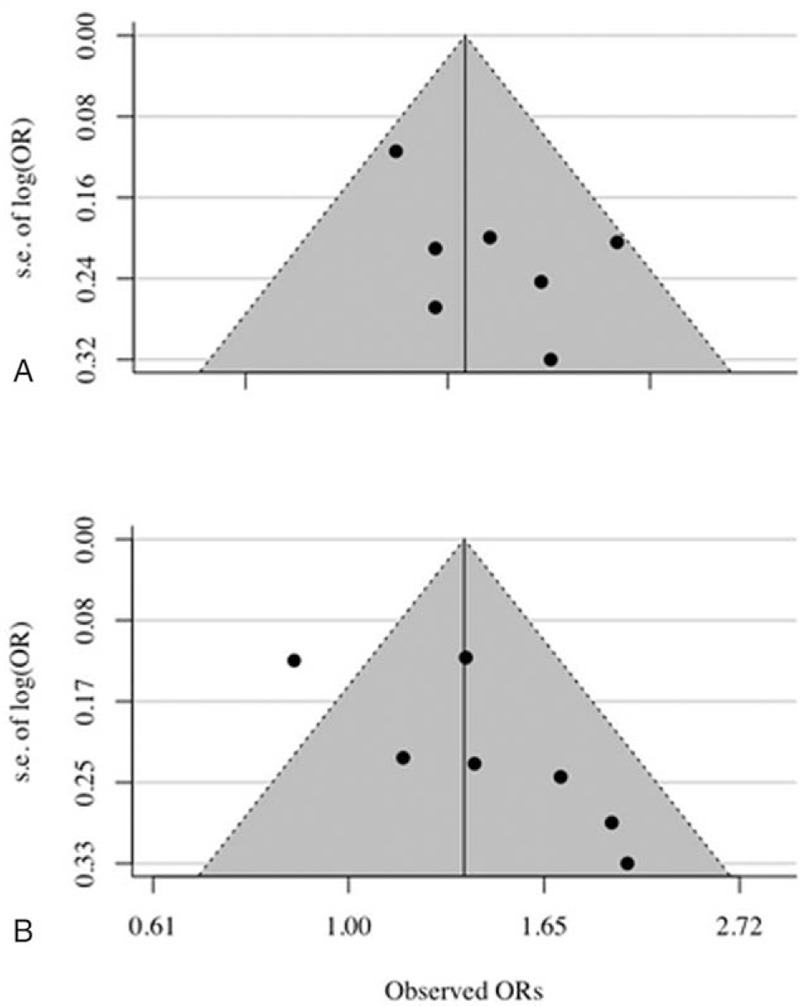
Funnel plot for analysis results of publication bias. (A) The second tertile of CRP level; (B) the third tertile of CRP level. CRP = C-reactive protein.

There was no evidence of publication bias as demonstrated by Begg's test (*P* = .56) and Egger's test (*P* = .12), and the near-symmetric funnel plot for the second tertile of CRP (Fig. 5A). However, the Ose and colleagues study may have introduced potential publication bias upon evaluation of the risk of the third tertile CRP level (Begg's *P* = .07 and Egger's *P* = .088, respectively) (Fig. 5B).^[15]^

## 4 Discussion

Our meta-analysis assessed the relationship between CRP levels and ovarian cancer risk. Overall, we identified a positive association between elevated levels of CRP and an increased risk of ovarian cancer. The overall findings demonstrated an estimated 34% increased risk of ovarian cancer when comparing women in the top tertile with women in the bottom tertile. Interestingly, the risk was even stronger in women with very high CRP levels (>10 mg/L).

Inflammation has long been proposed to play a role in the etiology of ovarian cancer.^[21]^ The hypothesis is based on the observations linking conditions associated with inflammation such as ovulation, pelvic inflammatory disease, endometriosis, and polycystic ovary syndrome with increased ovarian cancer risk.^[5,6,22–24]^ Consistent with this hypothesis, factors that reduce ovarian inflammation by inhibiting exogenous and endogenous irritants affecting the ovaries (e.g., tubal ligation, hysterectomy, or the use of NSAIDS) may offer protection against ovarian cancer.^[25–30]^ The acute-phase reactant C-reactive protein is a member of the pentraxin protein family and is a definitive biomarker of systemic inflammation.^[31]^ CRP is commonly used to evaluate the severity of systemic inflammation and outcomes of a variety of inflammation-related disorders.^[32]^ Normally, more than 70% of the population has a low CRP concentration (<0.3 mg/dL), whereas serum CRP levels in cancer patients is significantly higher.^[10,33]^ A plausible explanation is that tumor cells secrete a series of cytokines that strongly stimulate CRP production in the liver.^[34,35]^ Herein, serum CRP levels of cancer patients could be an indirect indicator of cancer-related inflammation.

Few epidemiological studies have focused on the association between CRP levels and ovarian cancer risk. A recent meta-analysis including 5 prospective studies reported a significant positive association between the CRP level and the ovarian cancer risk, with 995 cases and moderate heterogeneity between studies (*I*^2^ = 66.0%).^[14]^ Since then, 2 large-scale prospective studies have been published, adding 903 cases and 1646 controls to the evidence.^[15,19]^ Therefore, this updated systematic review and meta-analysis expanded the number of ovarian cancer cases evaluated to 1898 and the summary risk estimate revealed reduced heterogeneity (*I*^2^ = 59.39%) in contrast to the prior study.

Results from subgroup analysis showed that the country of origin, CRP assay methodology, CRP markers, sample sources and adjustment variables of age, smoking, NSAIDs use, HT use and OC use might be possible sources of heterogeneity. Despite considering the limitation of subgroup analysis to explain heterogeneity and the above variables were not recognized as precise sources of heterogeneity amongst studies, a few findings from the subgroup analysis warrant noting. The highest combined OR in the CRP level was found in the sample source from serum, which revealed that the differences of sample sources might confer the association seen between elevated levels of CRP and an increased risk of ovarian cancer. However, it might in turn suggest that measuring CRP from serum is a more sensitive approach compared with that from plasma. Similarly, Hs-CRP, as an inflammatory biomarker, may be more sensitive than common CRP in predicting ovarian cancer risk. Furthermore, our results demonstrated that smoking, NSAIDs use, HT use, and OC use might be influential factors for the ovarian cancer risk. As the largest prospective study to date, the Ose and colleagues study found no overall association between the CRP level and an elevated risk of ovarian cancer with the exception of women with increased CRP concentrations greater than 10 mg/L, which might be an important influential factor for the heterogeneity seen in the sensitivity analysis. Further accumulation of large-scale studies is anticipated to help improve the precision of the estimation and provide more comprehensive evaluation of these issues.

On evaluating findings from this meta-analysis, several limitations should be considered. First, heterogeneity existed among the studies in the meta-analysis, including different countries of origin, CRP diagnosis methods, CRP markers, smoking or parity status, NSAIDs use, HT use, and OC use. Therefore, the random-effects model meta-analysis was used to combine data whenever significant heterogeneity was found. Furthermore, sensitivity and subgroup analyses were performed to investigate potential sources of heterogeneity and appropriate inclusion criteria used to maximize homogeneity. Second, the number of studies included in this meta-analysis is relatively small. This resulted from performing a comprehensive and carefully literature search to identify relevant articles with results stratified by adjusted variables of BMI and parity status as not all studies involved here provided relevant information. Third, cancer is a heterogeneous disease and different histological types and clinical staging may influence the treatment and prognosis. Limited by the finite data, the analysis for the association between CRP levels and ovarian cancer risk stratified by histological type and staging could not be performed.

In summary, our findings supported a positive association between elevated CRP levels and an increased risk of ovarian cancer. These results indicated a role of chronic inflammation in ovarian carcinogenesis. Further studies, especially stringent and large-scale case-control studies are needed to definitively identify CRP as a direct player in ovarian carcinogenesis.
